# An Exploratory Study on Information Manipulation by Doctors: Awareness, Actual State, and Ethical Tolerance

**DOI:** 10.3390/clinpract12050075

**Published:** 2022-09-08

**Authors:** Shoichi Maeda, Eisuke Nakazawa, Etsuko Kamishiraki, Eri Ishikawa, Maho Murata, Katsumi Mori, Akira Akabayashi

**Affiliations:** 1 Department of Medical Ethics and Patient Safety, Keio University Keio Research Institute at SFC, 4411 Endo, Fujisawa 252-0883, Japan; 2 Course for Health Care Management and Public Health, Graduate School of Health Management, Keio University, 4411 Endo, Fujisawa 252-0883, Japan; 3 Department of Biomedical Ethics, Faculty of Medicine, University of Tokyo, 7-3-1 Hongo, Bunkyo-ku, Tokyo 113-0033, Japan; 4 Graduate School of Welfare and Health Sciences, Oita University, 700 Dannoharu, Oita 870-1192, Japan; 5 Comprehensive Research Organization, Institute for Global Health, Waseda University, 1-3-10 Nishiwaseda Shinjyuku-ku, Tokyo 169-0051, Japan; 6 Department of Clinical Oral Oncology, Graduate School of Biomedical Sciences, Nagasaki University, Sakamoto1-7-1, Nagasaki 852-8588, Japan; 7 Division of Medical Ethics, School of Medicine, New York University, 227 East 30th Street, New York, NY 10016, USA

**Keywords:** informed consent, information manipulation, truth-telling, prognosis disclosure, patient-doctor relationship, trust, Japan

## Abstract

(1) Background: To what extent is information manipulation by doctors acceptable? To answer this question, we conducted an exploratory study aimed at obtaining basic data on descriptive ethics for considering this issue. (2) Methods: A self-administered questionnaire survey was conducted on a large sample (*n* = 3305) of doctors. The participants were queried on (1) whether they consider that information manipulation is necessary (awareness), (2) whether they have actually manipulated information (actual state), and (3) their ethical tolerance. (3) Result: The response rate was 28.7%. Sixty percent of the doctors responded that information manipulation to avoid harm to patients is necessary (awareness), that they have actually manipulated information (actual state), and that information manipulation is ethically acceptable. (4) Conclusion: While the present survey was conducted among doctors in Japan, previous studies have reported similar findings in the United States and Europe. Based on our analysis, we hypothesize that a relationship of trust between patients and medical personnel is crucial and that information manipulation is not needed when such a relationship has been established.

## 1. Introduction

The problem of clinical communication, represented by truth-telling, is one of the classic issues of medical ethics. Prognosis disclosure is an important issue with reference to information manipulation by doctors [1,2,3,4]. There appears to be a gap between patient awareness and actual information manipulation by doctors. According to a classic survey conducted between November 2004 and April 2005 among oncologists in the United States (*n* = 1137) [1], only 42% of doctors constantly communicated with patients about their prognosis, and 48% of the doctors disclosed prognosis only when requested by patients. In addition, 57% of the doctors did not necessarily provide a specific time frame pertaining to the prognosis.

Unfavorable news may cause psychological harm to patients. When providing medical information, the extent of paternalistic manipulation of information in consideration of the nonmaleficence principle seems to be largely at the discretion of doctors even today. It has often been pointed out that cultural differences underlie this phenomenon. In 2011, O’Kelly commented on diagnosis and prognosis, focusing primarily on cases in the Middle East. Diagnosis and prognosis tend to be concealed in the Middle East and Asia, which is based on the consideration of avoiding psychological harm to patients [5]. On the other hand, although there are differences among cultures, many patients desire to be notified of unfavorable news [6,7]. Miyata et al. reported a similar finding in a study including 440 doctors [8]. However, despite the pervasive idea of self-determination in society, it has been reported that the practice of entrusting important health decisions to medical personnel and family remains prevalent. Akabayashi et al. reported these findings in a study including 74 university hospital doctors [9]. There is also a report of a case study that illustrated entrusting health decisions to medical personnel and family in the United States [10]. As reported above [1], doctors manipulate information on the prognosis of patients occurs in the United States, where the principles of respecting one’s autonomy and self-determination are highly emphasized; this practice has also been reported to occur in European countries such as Italy (*n* = 1271) and Sweden [11,12].

Ethics regarding prognosis disclosure has always been a point of active discussion [13], but the depth of the discussion has been insufficient [3]. Ethical discussion on information manipulation by doctors is still often conducted based on considerations of patients’ voluntary self-determination of treatment decisions and nonmaleficence [14]. Regarding patient self-determination, whether a doctor provides information to a patient depends on how the patient desires the information to be provided. All information should be disclosed to a patient if he/she desires it. However, if the patient does not desire that all information be provided, a doctor should not disclose all medical information to the patient based on the ‘right not to know’. In this context, Stahl and Tomlinson (United States) presented a prognosis disclosure policy based on Kantian autonomy in 2017 [15]. This policy was revolutionary in the context of previous discussions on balancing considerations of self-determination and psychological harm. However, they claimed that “the truth should be told, even if it is not desired by patients.” This Kantian autonomy approach has drawbacks in terms of consistency with the practice of prognosis disclosure in healthcare settings and support by intuition.

To what extent is information manipulation by doctors acceptable? We define information manipulation as “not to disclose the truth at the discretion of the physician for the benefit of the patient.” So, ethically speaking, it is an action based on the principle of beneficence and nonmaleficence. The dissociation between the claims of ethicists and the actual state of medical practice in the United States indicates that this issue **is not necessarily culturally dependent**. Therefore, we conducted a **large-scale questionnaire survey** on (1) whether doctors consider that information manipulation is necessary (awareness), (2) whether they have actually manipulated information (actual state), and (3) their ethical tolerance. This exploratory study was aimed at obtaining basic data on descriptive ethics for considering these issues.

## 2. Methods

Study Design: anonymous self-administered questionnaire. In February 2014, we conducted an anonymous self-administered questionnaire survey by e-mail.

Study population: Member of the Japan Medical Association and doctors responsible for clinical training at basic clinical training hospitals and university hospitals throughout Japan. Overall, 2291 doctors with all specialties were chosen by randomly selecting 2.5% and 10% of the members of the Japan Medical Association, whose primary medical department was internal medicine and surgery, respectively. We also included doctors responsible for clinical training at all basic clinical training hospitals and university hospitals (total, *n* = 3305).

Variables: The items used in the questionnaire.

The items used in the questionnaire. The seven researchers, including physicians, nurses, legal scholars, and ethicists, discussed and created the questionnaire based on previous studies. In particular, since previous studies have shown that manipulation of information by healthcare professionals is a problem (e.g., 2008 Journal of Clinical Oncology: JCO), the questions were designed to focus on information manipulation. First, to investigate the current situation in Japan, we conducted a highly representative sampling and asked a large number of physicians (*n* = 3305) about their awareness and actual practice. Prior to implementation, 10 physicians were pretested and asked their opinions, and feedback was received that the questions were easy to answer and important. The four short questions can be answered in less than five minutes.

“Regarding explanation by manipulating information with good intentions” (hereinafter referred to as “explanation with manipulation”) with reference to the explanation provided while obtaining informed consent at the time of medical treatment (hereinafter referred to as “explaining informed consent”). In the “explanation with manipulation,” explanation by manipulating information with good intentions was first defined as giving a false explanation (such as describing a malignant disease as a benign disease to a patient to eliminate his/her anxiety), an understated explanation (such as understating the degree of side effects to a patient to encourage continued medication), or an exaggerated explanation (such as overstating the degree of exacerbation of a disease to a patient to encourage medical attention). Then, respondents were asked whether “explanation with manipulation is necessary in some cases,” with two possible answers of “Agree” and “Disagree.” Furthermore, participants were asked whether they “have provided an explanation with manipulation,” with two possible answers of “Yes” and “No,” and whether “explanation with manipulation is ethically acceptable,” with two possible answers of “Agree” and “Disagree.” Additionally, they were asked whether they “request a nurse to be present ‘at the time of informed consent’” (hereinafter referred to as “the presence of a nurse”), with two possible answers of “Yes” and “No.” In addition, doctors were asked to provide information on their attributes, including sex, age, primary specialty, and employing medical institutions. At ‘the time of informed consent’ means when the attending physician explains the diagnosis and treatment options with the physician’s recommendation, inviting patient (and family’s) questions. After enough conversation, when the patient has decided to choose one treatment option, the patient signs the document of agreement with the treatment option. When the patient cannot decide at that point, the physician stops the process and gives more time to the patient unless the case is an emergency.

Data collection: By replying through e-mail.

Data analysis and Statistical: For the analysis, the three items related to “explanation with manipulation” and “the presence of a nurse” were grouped by the attributes of doctors, the type of their employing medical institutions, the number of hospital beds, and their primary specialty. First, we examined whether there were differences between the groups in awareness, action, and ethical views. Then, we examined whether there were differences in the proportion of those who request “the presence of a nurse” depending on the awareness and action regarding “explanation with manipulation.” Lastly, the association between the age of the doctors and the number of hospital beds in their employing medical institutions was examined. The χ^2^ test was used for all statistical tests. Statistical analysis was performed using SAS version 9.4 (SAS Institute Inc., Cary, NC, USA), and *p* < 0.05 was considered statistically significant.

### Ethical Consideration

This study was conducted with the approval of the Research Ethics Review Committee of Yamaguchi Prefectural University.

## 3. Results

Questionnaires were sent to 3305 doctors, and 946 responded. In the case of 14 potential participants, the questionnaires were returned due to “unknown address”; these potential participants were excluded from the study. Thus, the response rate was 28.7%. A total of 946 valid responses were obtained in this study. Data is contained within the Supplementary Materials.

The attributes of the respondents and their employing medical institutions are shown in Table 1. The majority of the respondents were men, and many were in their 40s and 50s. With regard to the employing medical institutions, clinical training hospitals other than university hospitals accounted for approximately half, and many were medium-scale hospitals with 200–499 beds.

Of the respondents who provided valid responses (*n* = 946), 546 (57.7%) answered that information manipulation was necessary, 622 (65.8%) had manipulated information, and 609 (64.4%) considered that information manipulation was ethically acceptable. This indicates that approximately 60% of the doctors responded that information manipulation was necessary, that they had manipulated information, and that information manipulation was ethically acceptable; all the responses provided complete information pertaining to the three components of awareness, actual state, and ethical tolerance for “explanation with manipulation”.

The responses regarding “the presence of a nurse” and the awareness, actual state, and ethical tolerance for “explanation with information manipulation” were grouped by sex, age, and primary specialty of respondents, as well as the type of their employing medical institutions and the number of hospital beds. The results are shown in Table 2.

Regarding the proportion of those who “request a nurse to be present at the time of explaining informed consent,” there was no statistically significant difference among groups delineated based on respondents’ attributes of sex, age, and primary specialty. However, a statistically significant difference was seen with respect to the type of medical institutions and the number of hospital beds. The proportion of those who requested a nurse to be present was significantly larger among doctors working in clinics with less than 20 beds or university hospitals than those working at small or medium-scale hospitals. In addition, there was a statistically significant difference in the proportion of those who “consider that explanation with manipulation is necessary in some cases” depending on sex and age; the proportion was significantly smaller among women than among men and among doctors over the age of 60 than among those below 60 years of age. Furthermore, a statistically significant difference was seen in the proportion of those who “have manipulated information” depending on age and the number of hospital beds at the medical institutions. With regard to age, the proportion of those who “have manipulated information” was significantly smaller in doctors over the age of 60 years than among those aged less than 60 years. With regard to the number of hospital beds, the proportion of those who “have manipulated information” was significantly smaller among doctors working at clinics with less than 20 beds or at large-scale hospitals with 500 or more beds than among doctors working at small or medium-scale hospitals. Furthermore, a statistically significant difference was observed in the proportion of those who “consider that explanation with manipulation is ethically acceptable” with respect to sex and age; the proportion was significantly smaller among women than among men and among doctors over the age of 60 years than among those aged less than 60 years.

Table 3 shows the proportions of those who request “the presence of a nurse” depending on the awareness and action associated with “explanation with manipulation.” In response to any of the questions on whether they consider that explanation with manipulation is necessary in some cases, whether they have manipulated information, and whether they consider that explanation with manipulation is ethically acceptable, more than 60% of respondents responded that they “request a nurse to be present at the time of explaining informed consent”; there was no statistically significant difference among those who agreed and those who disagreed with the questionnaire items.

Lastly, the association between the age of the respondents and the number of hospital beds at their employing medical institutions is shown in Table 4. There was a significant association between the age of the doctors and the number of hospital beds at their employing medical institutions. While approximately 20% of the doctors under the age of 60 worked at clinics with less than 20 beds, approximately half of the doctors over the age of 60 worked at clinics with less than 20 beds each.

## 4. Discussion

### 4.1. Information Manipulation by Doctors Is Clearly Prevalent

Unlike a previous study targeting only oncologists [1] or studies targeting the general public [16], the present survey targeted doctors more broadly throughout Japan by including a sample not limited by specialty. Analysis of the previous study states that: ‘*Although 98% said their usual practice is to tell terminally ill patients that they will die, 48% specifically described communicating terminal prognoses to patients only when specific preferences for prognosis information were expressed. Forty-three percent said they always or usually communicate a medical estimate of time as to when death is likely to occur, and 57% reported sometimes, rarely, or never giving a time frame* [1]*, 69.6%) respondents agreed to follow the doctor’s discretion, whilst 111 (66.1%) respondents agreed to follow the family member’s decision. For respondents who preferred to have the diagnosis and prognosis withheld, 59 (26.5%) agreed to follow the doctor’s decision, and 79 (35.3%) of respondents agreed with following family member’s wishes* [16].’ These suggested that the physicians hesitate to tell the diagnosis to the patients in the USA [1], and patients do not want to decide their treatment options by themselves, rather entrusting physicians or family, at least in Japan [16].

Our data revealed the awareness and actual state of information manipulation by doctors. The doctors responded that information manipulation is necessary, that they have actually manipulated information, and that information manipulation is ethically acceptable. We speculate that the analysis of their responses may indicate that they have manipulated information for the benefit of patients in matters such as “describing a malignant disease as benign to a patient to eliminate his/her anxiety” and “understating the degree of side effects to a patient to encourage continued medication,” indicating the strong paternalism of doctors. The ethical position in support of this practice includes the principles of nonmaleficence (however, this stance is criticized by self-determinationists). This is what we have done, and this is what we have found.

### 4.2. Do Nurses Have the Same Viewpoint?

This study showed unexpected results regarding the presence of a nurse at the time of explaining informed consent. We initially thought that doctors would rarely request a nurse to be present when manipulating information because they would not like the nurses to know about information manipulation or would like to avoid subsequent criticism. However, the results showed no significant difference in the request for the presence of a nurse between those who manipulated information and those who did not (Table 3). We speculate that this result may be interpreted as follows. Explaining informed consent in the presence of a nurse is recommended from the viewpoint of team medicine, and the fact that 60% of the doctors requested the presence of a nurse is considered favorable. In fact, the proportion of those who requested a nurse to be present was significantly large among doctors in training hospitals due to educational considerations (Table 2). However, the presence of a nurse when a doctor manipulates information while explaining informed consent may imply that the nurse may also accept the explanation. If this interpretation is partially valid, it indicates that nurses do not necessarily oppose paternalistic information manipulation by doctors. Therefore, some nurses may consider that information manipulation for the benefit of patients is acceptable to some extent. For example, if a nurse who frequently interacts with patients on a daily basis and values patient care knows that a patient is highly anxious and mentally weak, he/she may understand the need for information manipulation by doctors to reduce the anxiety of the patient. (It should be noted that the present study alone cannot clarify whether nurses present at the time of explaining informed consent subsequently criticized the doctors who manipulated information; doctors and nurses may not hold equal positions in their relationship, making it difficult for nurses to criticize doctors. As this is simply speculation of the authors, further detailed studies targeting nurses will be needed).

However, having a mindset of ‘for the patient good’ may motivate healthcare workers in general to take action [17]. For example, although paternalism should be rejected in many cases, healthcare workers who rushed to do their duty during the COVID-19 pandemic or a catastrophe (for patients) without regard for personal danger have been praised as virtuous. So as to avoid misunderstanding the intentions of the doctors, it should be noted that we are not advocating “information manipulation” itself. However, we claim that ‘for the patient good’ may be an essential quality of conscientious healthcare workers.

### 4.3. Sex Differences

Comparing male and female doctors, the perceived necessity and ethical tolerance for information manipulation were significantly lower in female doctors. It is difficult to interpret this finding because of the small number of female respondents and the scarce information. However, it should be noted that issues of sex will become even more important in the coming era.

### 4.4. Does a Relationship of Trust Reduce Such Paternalism?

Interestingly, our results showed that the proportion of those who consider that information manipulation by doctors is necessary, who have actually manipulated information, and who consider that information manipulation is ethically allowed was significantly smaller among doctors over the age of 60 than among younger doctors (Table 2). To understand the reason underlying this observation, we compared data regarding the age of doctors and the number of hospital beds (Table 4). Our analysis showed that more than half of doctors over the age of 60 worked at hospitals with less than 20 beds (statistically significant). In the following passage, we speculate upon the possible reason for this finding (we believe such speculation is acceptable as this is an exploratory study).

Many older doctors who provide medical care in clinics with less than 20 beds each are doctors in private practice who are closely attached to the community. In such a medical setting, the relationship between the patients and doctors is sufficiently established, and those doctors likely have given medical consultations to many patients with chronic diseases for a long period of time. Such an environment differs from the complex and difficult medical practice situations at large hospitals. At smaller clinics, the older doctors are familiar with the personalities of the patients and their families, and they likely often take sufficient time to explain medication adherence and other issues, gaining the understanding of the patients. Furthermore, due to the relationship of trust between individual doctors and patients/family members, clinics employ a small number of staff members and do not particularly require team medicine; this likely reduces the need for a nurse to be present when the doctors explain informed consent to the patients. Therefore, an established relationship of trust may reduce the need for unnecessary paternalism, creating an environment in which unfavorable news can be openly discussed. These results have led us to hypothesize that such a relationship of trust between patients and medical personnel is important and that establishing such a relationship may eliminate the need for information manipulation.

### 4.5. Limitations

(1) Low response rate (however, compared to previous studies, the present study is a large-scale sampling and is superior as it targets not only oncologists but also doctors from all medical departments).
(2) Questionnaire development and standardization have not been completed. Validity and reliability have not been examined. However, an expert in empirical surveys commented: *This is an exploratory fact-finding and awareness survey. It is not a scale development such as psychological questionnaires. Standardization, evaluation its validity and reliability are not necessary, rather it is impossible. So, your description of the methods is enough*.
(3) Limitations of a cross-sectional questionnaire survey conducted only once in Japan. Limitations were pertinent to an exploratory study.
(4) The study targets only doctors (other healthcare workers such as nurses were not included).
(5) A small number of question items (a text box providing an opportunity for the respondents to explain or provide further information could have been included in the questionnaire).
(6) Several discussions have been presented to explain the study findings. The explanations are purely speculatory as this is an exploratory study with a small number of questions (resulting in a small amount of data). Studies to test the hypothesis need to be conducted in the future.

Despite these limitations, this exploratory study is of great significance as it has led to a plausible hypothesis on this topic. Moreover, as the problem of the discretionary power of doctors in information manipulation is a critical issue internationally, the present study may contribute to comparative and international collaborative studies.

## 5. Conclusions

We surveyed doctors throughout Japan regarding their awareness, actual state, and ethical tolerance of the need for information manipulation at the time of explaining informed consent. More than 60% of the respondents answered that information manipulation is necessary, that they have actually manipulated information, and that information manipulation is ethically acceptable. Although the results appear to suggest the rampant paternalism of doctors, we speculate that information manipulation may be carried out from the ethical standpoint of ‘for the patient good.’ Lastly, the results of this study suggested that it is important to build a relationship of trust between patients and doctors in small-scale medical settings such as clinics and that information manipulation may not be needed in an environment in which such a relationship of trust has been established. These findings suggest that information manipulation by doctors is not necessarily due to paternalism but involves various factors. Therefore, further ethical discussion on information manipulation by doctors is needed worldwide.

## Figures and Tables

**Table 1:** Attributes of the respondents and their employing medical institutions.

		Number of Respondents (%)	
Sex	Male	888	(94.5%)
	Female	52	(5.5%)
Age	20–39 years	26	(2.8%)
	40–59 years	582	(61.6%)
	Over 60 years	337	(35.7%)
Area	Hokkaido/Tohoku	135	(14.3%)
	Shinetsu/Hokuriku	63	(6.7%)
	Kanto	226	(23.9%)
	Tokai	112	(11.9%)
	Kinki	159	(16.8%)
	Chugoku/Shikoku	119	(12.6%)
	Kyushu/Okinawa	131	(13.9%)
Medical institution	University hospitals	70	(7.4%)
	Clinical training hospitals other than university hospitals	433	(46.0%)
	Hospitals other than the above	154	(16.4%)
	Clinics	284	(30.2%)
Number of hospital beds	Less than 19 beds	285	(31.5%)
	20–199 beds	115	(12.7%)
	200–499 beds	330	(36.5%)
	500 or more beds	175	(19.3%)
Primary specialty	Internal medicine	510	(54.3%)
	Surgery	309	(32.9%)
	Others	121	(12.9%)

**Table 2:** The presence of a nurse and the awareness and action regarding explanation with manipulation by attribute and employing medical institution.

		“I Request a Nurse to Be Present at the Time of Explaining Informed Consent.”			“I Think that Explanation with Manipulation Is Necessary in Some Cases.”		
		Number of Respondents (%)		*p*-Value ^†^	Number of Respondents (%)		*p*-Value ^†^
Sex	Male	557	(64.6%)	0.101	523	(59.8%)	0.038
	Female	26	(53.1%)		23	(45.1%)	
Age	20–39 years	13	(50.0%)	0.764	18	(69.2%)	<0.001
	40–59 years	370	(64.9%)		370	(63.8%)	
	Over 60 years	204	(63.8%)		162	(49.8%)	
Medical institution	University hospitals	36	(55.4%)	<0.001	38	(54.3%)	0.214
	Clinical training hospitals other than university hospitals	300	(69.6%)		255	(59.0%)	
	Hospitals other than the above	104	(68.9%)		100	(66.2%)	
	Clinics	146	(54.9%)		156	(56.7%)	
Number of hospital beds	Less than 19 beds	146	(54.7%)	<0.001	155	(56.2%)	0.061
	20–199 beds	84	(73.7%)		78	(69.0%)	
	200–499 beds	234	(71.6%)		204	(62.2%)	
	500 or more beds	100	(58.8%)		98	(56.0%)	
Primary specialty	Internal medicine	306	(61.9%)	0.095	306	(61.2%)	0.157
	Surgery	193	(64.1%)		179	(58.5%)	
	Others	85	(72.6%)		62	(51.7%)	
		**“I have given an explanation with manipulation.”**			**“I think that explanation with manipulation is ethically acceptable.”**		
		**Number of respondents (%)**		***p*-Value ^†^**	**Number of respondents (%)**		***p*-Value ^†^**
Sex	Male	593	(67.7%)	0.110	582	(66.8%)	0.042
	Female	29	(56.9%)		27	(52.9%)	
Age	20–39 years	18	(69.2%)	<0.001	18	(69.2%)	0.013
	40–59 years	420	(72.4%)		399	(69.4%)	
	Over 60 years	188	(57.7%)		195	(59.8%)	
Medical institution	University hospitals	46	(65.7%)	0.144	43	(62.3%)	0.424
	Clinical training hospitals other than university hospitals	299	(69.2%)		280	(65.1%)	
	Hospitals other than the above	109	(71.7%)		109	(71.7%)	
	Clinics	171	(62.2%)		179	(65.6%)	
Number of hospital beds	Less than 19 beds	171	(62.0%)	0.006	178	(65.0%)	0.234
	20–199 beds	84	(73.7%)		84	(73.7%)	
	200–499 beds	241	(73.5%)		221	(67.6%)	
	500 or more beds	111	(63.4%)		109	(62.6%)	
Primary specialty	Internal medicine	337	(67.3%)	0.271	325	(65.3%)	0.547
	Surgery	211	(69.0%)		208	(68.0%)	
	Others	73	(60.8%)		74	(62.7%)	

†: Pearson’s χ^2^ test.

**Table 3:** The presence of a nurse by awareness and action with reference to explanation with manipulation.

	“I Require a Nurse to Be Present at the Time of Explaining Informed Consent”		
	Number of Respondents (%)		*p*-Value ^†^
“Explanation with manipulation is necessary in some cases”			
Agree	341	(63.4%)	0.549
Disagree	243	(65.3%)	
“I have given an explanation with manipulation”			
Yes	398	(65.1%)	0.353
No	186	(62.0%)	
“Explanation with manipulation is ethically acceptable”			
Agree	379	(63.2%)	0.452
Disagree	203	(65.7%)	

†: Pearson’s χ^2^ test.

**Table 4:** Age of the respondents and the number of hospital beds in their employing medical institutions.

Age	Less than 19 Beds		20–199 Beds		200–499 Beds		500 or More Beds		*p*-Value ^†^
20–39 years	6	(23.1%)	8	(30.8%)	7	(26.9%)	5	(19.2%)	<0.001
40–59 years	116	(20.9%)	72	(12.9%)	249	(44.8%)	119	(21.4%)	
Over 60 years	162	(50.3%)	35	(10.9%)	74	(23.0%)	51	(15.8%)	

†: Pearson’s χ^2^ test.

## Data Availability

Data is contained within the Supplementary Materials.

## Supplementary Materials

The following supporting information can be downloaded at: https://www.mdpi.com/article/10.3390/clinpract12050075/s1.
